# Permanent Pacemaker Placement Secondary to Remdesivir Induced Bradycardia: A Case Report

**DOI:** 10.7759/cureus.30923

**Published:** 2022-10-31

**Authors:** Shahkar Khan, Ahmad Mustafa, Sherif M Elhosseiny, Taqi Rizvi, James Lafferty

**Affiliations:** 1 Internal Medicine, Staten Island University Hospital, Staten Island, USA; 2 Cardiology, Staten Island University Hospital, Staten Island, USA

**Keywords:** remdesivir, covid-19, av block, cardiac pacemaker, severe bradycardia

## Abstract

Remdesivir is approved by the FDA for the treatment of hospitalized coronavirus disease 2019 (COVID-19) patients. It is known to be associated with transient bradycardia that resolves after discontinuation of the drug. We present a case of a 71-year-old male with a history of congestive heart failure, hypertension, and atrial flutter (rate controlled with carvedilol) presented for evaluation of worsening dyspnea, dry cough, and fatigue. His COVID-19 reverse transcription-polymerase chain reaction (RT-PCR) was positive and his chest x-ray showed right mid-lung opacity. Oxygen saturation was 88% on room air. He was started on dexamethasone and remdesivir. Bradycardia was noted on telemetry monitoring 48 hours after starting remdesivir. Carvedilol was discontinued, but the bradycardia persisted. Heart rate reached a nadir of 38 beats per minute (bpm) three days after completion of remdesivir therapy. Due to persistent bradycardia, he received a dual-chamber cardiac pacemaker without any immediate complications. Three months later, his pacemaker interrogation showed 99% ventricular pacing. We recommend that extra caution should be taken when initiating remdesivir therapy in individuals with baseline conduction abnormalities due to the possibility of persistent bradycardia.

## Introduction

The emergence of coronavirus disease 2019 (COVID-19) has had widespread implications on healthcare. The scientific community responded to this new threat with extensive research that helped identify new medications and therapeutic approaches to combat this deadly disease. The current medications available include and are not limited to remdesivir, tocilizumab, Paxlovid, and dexamethasone among others. The non-medication interventions that can be used include hyperbaric oxygen therapy and extracorporeal membrane oxygenation (ECMO) [1]. One of the pharmaceutical tools available to combat COVID-19 is remdesivir, which is metabolized into a nucleoside analog which is then incorporated into the viral RNA leading to the inhibition of viral replication [2]. The consensus among the clinical community is that remdesivir treatment should be started as soon as possible in hospitalized patients to slow the progression of the disease and prevent complications [3]. The emergency authorization by the FDA led to the widespread use of remdesivir, and further studies have validated the effectiveness of remdesivir therapy [3-5].

Commonly reported side effects of remdesivir include, transaminitis, nausea, hypokalemia, and hypersensitivity reactions [6]. Furthermore, there have been numerous case reports stating bradycardia as a side effect of remdesivir treatment. In all but one of these cases, the bradycardia resolved after remdesivir was stopped [6,7]. We present a case of persistent bradycardia in a patient with history of chronic atrial flutter with variable atrioventricular (AV) block. The bradycardia persisted even after stopping remdesivir, which necessitated the implantation of a permanent pacemaker.

## Case presentation

**Figure 1:**
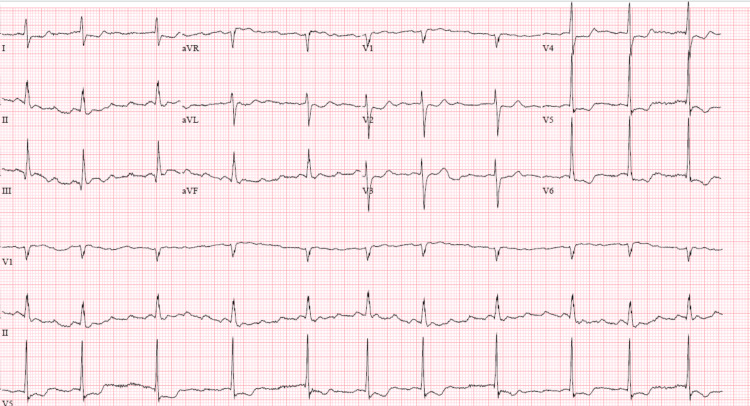
EKG showing baseline atrial flutter with variable AV block. AV: atrioventricular

A 71-year-old male presented for evaluation of worsening dyspnea, dry cough, and fatigue. His past medical history included heart failure with preserved ejection fraction, coronary artery disease status-post coronary artery bypass grafting, atrial flutter, and hypertension. His family history included myocardial infarction in his father, mother, and one sibling. He was an active smoker and averaged approximately half a pack per day for more than 60 years. He was recently discharged from the hospital after being treated for heart failure exacerbation. His medications included carvedilol for heart failure and rate control of atrial flutter. Physical examination revealed coarse breath sounds, an irregular heart rate, and 1+ lower extremity edema. Vital signs showed an oxygen saturation of 88% on room air. He was initially placed on bilevel positive airway pressure (BiPAP) and was then transitioned to a nasal cannula. His EKG was consistent with atrial flutter with variable atrioventricular block (Figure 1).

His COVID-19 reverse transcription-polymerase chain reaction (RT-PCR) was positive significant laboratory values (Table 1). The patient’s chest x-ray showed right mid-lung opacity. His echocardiogram showed an ejection fraction of 64%. The aortic valve gradients and size were consistent with mild aortic stenosis. The serum electrolytes were within normal limits.

**Table 1:** Pertinent lab values on admission and early during the hospital course.

Parameter	Values	Reference range
Hemoglobin	6.2 g/dL	14-18 g/dL
White blood cell count	7.98 K/uL	4.8-10.8 K/uL
Platelet count	303 K/uL	130-400 K/uL
Lactate dehydrogenase	347 U/L	50-242 U/L
Ferritin	490 ng/mL	30-400 ng/mL
Procalcitonin	2.67 ng/mL	0.02-0.10 ng/mL
Creatine	3.0 mg/dL	0.7-1.5 mg/dL
Thyroid-stimulating hormone	0.98 uIU/mL	0.27-4.20 uIU/mL
C-reactive protein	48 mg/L	≤4 mg/L
D-dimer	327 ng/mL DDU	0-230 ng/mL DDU
Brain natriuretic peptide	8057 pg/mL	0-300 pg/mL
Troponin	0.24 ng/mL	≤0.01 ng/mL

**Figure 2:**
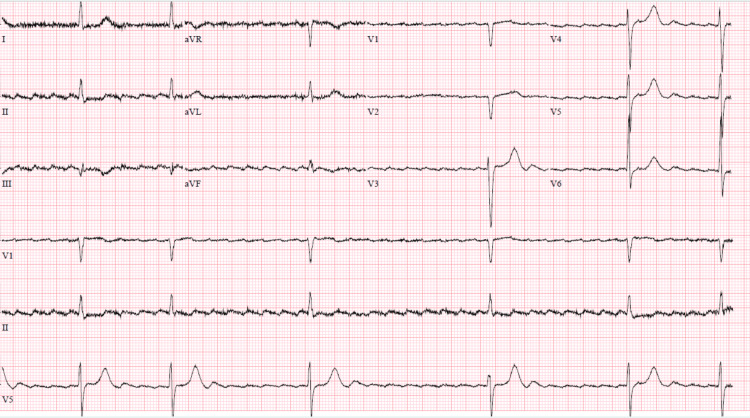
EKG with worsening high-grade AV block. AV: atrioventricular

The patient received one unit of packed red blood cells in the setting of demand ischemia. He also received IV Lasix 20 mg. He was treated for COVID-19 with a regimen of dexamethasone 6 mg IV once daily. Remdesivir was added on day two of admission with an initial dose of 200 mg IV on day one, followed by 100 mg IV daily for the next four days. Episodes of bradycardia were noted on telemetry monitoring 48 hours after starting remdesivir therapy. During these episodes, he remained asymptomatic. Carvedilol was initially reduced and eventually discontinued due to the persistence of bradycardia. His heart rate reached a nadir of 38 beats per minute (bpm) three days after completion of remdesivir therapy (Figure 2).

The average heart rate during the five-day remdesivir regimen was around 55 bpm, while the average heart rate for the five days following remdesivir treatment was 53 bpm. The patient was evaluated by the cardiac electrophysiologist for nine days of persistent bradycardia after completion of remdesivir therapy. His EKG also showed worsening high-grade AV block. He received a dual chamber cardiac pacemaker on hospital day 15 without any immediate complications and was discharged the following day.

## Discussion

There have been multiple trials evaluating the effectiveness of remdesivir for COVID-19 such as the WHO solidarity trial [8] and the Adaptive COVID-19 Treatment Trial-1 (ACTT-1) [9]. The WHO solidarity trial included 11,330 adults and found no difference in mortality between the different treatment groups. In the ACTT-1 trial, a total of 1,062 patients hospitalized with COVID-19 were randomized to receive either a placebo or remdesivir. The results of this trial showed that patients who received remdesivir had a shorter time to recovery [9].

The cardiotoxic effects of remdesivir have been well-reported even before the COVID-19 pandemic. Earlier trials that evaluated the efficacy of remdesivir therapy in the Ebola epidemic reported multiple cardiac side effects including cardiac arrest, hypotension, bradycardia, and atrial fibrillation [10]. With the widespread use of remdesivir in the treatment of COVID-19, these side effects have now been reported in COVID-19 patients as well [11]. The occurrence of bradycardia during treatment with remdesivir is also well-reported [6,7,12,13]. We reviewed a retrospective study that evaluated a cohort of 473 patients being treated with remdesivir [12]. The study showed that bradycardia (heart rate (HR)<60 beats/min) was observed in 1.3% of the patients before the initiation of treatment with remdesivir. However, the rate of bradycardia was directly proportional to the duration of remdesivir therapy, and on day five bradycardia was observed in 16.8% of the cohort [12]. Another study showed that the incidence of transient bradycardia in patients being treated with remdesivir can be as high as 47% [14]. Interestingly bradycardia during treatment was associated with more favorable outcomes [12,15]. We also reviewed a study that evaluated the cardiotoxic effects on cardiomyocytes derived from human pluripotent stem cells. This study reported an increased risk of QT prolongation [16]. The changes in the electrocardiogram induced by treatment with remdesivir are not only limited to bradycardia and QT prolongation as mentioned above, but they also include a rightward T-axis deviation [17]. The rightward T-axis deviation is an indicator of increased mortality risk [18].

The exact mechanism of remdesivir-induced bradycardia is unknown. However, the proposed mechanisms include the following: (1) the active metabolite of remdesivir is a nucleoside triphosphate which is similar to adenosine triphosphate (ATP) and ATP itself has negative chronotropic effects on the sinoatrial (SA) node through vagal stimulation [19]; (2) the ATP metabolite adenosine binds the AV node and induces a negative chronotropic effect [13,19]; and (3) the binding of remdesivir to mitochondrial RNA polymerase can lead to cardiotoxicity [20,21].

The bradycardia caused by remdesivir therapy is usually transient [22]. Bradycardia that persisted after stopping remdesivir has been reported in only one other case report [7]. However, unlike the other case report, this patient's bradycardia was severe and persistent enough to necessitate the placement of a permanent pacemaker.

**Figure 3:**
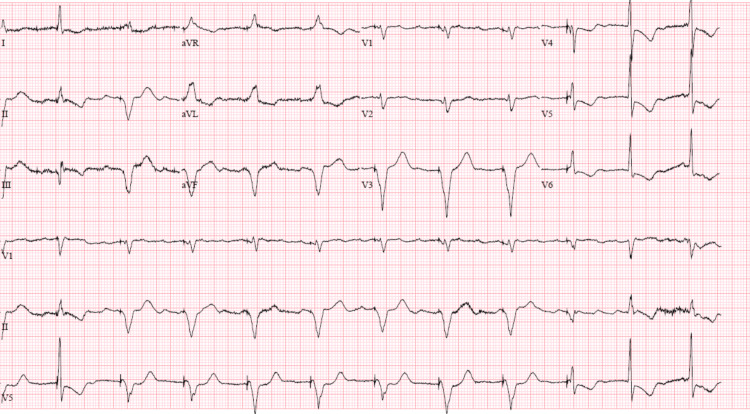
Post pacemaker EKG showing ventricular paced rhythm.

It is noteworthy to mention that he had an elevated troponin on admission that trended down after his sepsis and acute anemia were appropriately treated. He needed beta blockers for rate control at baseline, but following remdesivir therapy, he required a pacemaker due to bradycardia. To our knowledge, this is the first case of persistent bradycardia after the administration of remdesivir requiring the placement of a permanent pacemaker. The post permanent pacemaker EKG showed ventricular paced rhythm (Figure 3). In addition, his pacemaker interrogation revealed his dependency on the pacemaker as the total ventricular-paced (V-paced) time was 98.7%. This observation leads us to believe that remdesivir might have been responsible for a cardiac conduction abnormality that failed to resolve after stopping remdesivir. This permanent cardiac conduction abnormality can be explained by mitochondrial dysfunction and permanent damage to the conduction system induced by remdesivir treatment.

## Conclusions

Keeping the data presented in mind, we suggest that a baseline EKG should be done before the initiation of remdesivir therapy. Patients with baseline conduction abnormalities undergoing treatment with remdesivir therapy should have their cardiac rhythm continuously monitored with a telemonitor. Extra caution should be exercised in this patient population, and treatment with remdesivir should only be initiated after carefully weighing the risks and benefits of therapy. The addition of additional QT-prolonging medications should be avoided as well.

## References

[1] McFee RB (2020). COVID-19: therapeutics and interventions currently under consideration. Dis Mon.

[2] Eastman RT, Roth JS, Brimacombe KR, Simeonov A, Shen M, Patnaik S, Hall MD (2020). Remdesivir: a review of Its discovery and development leading to emergency use authorization for treatment of COVID-19. ACS Cent Sci.

[3] Aleissa MM, Silverman EA, Acosta LM, Nutt CT, Richterman A, Marty FM (2020). New perspectives on antimicrobial agents: remdesivir treatment for COVID-19. Antimicrob Agents Chemother.

[4] Spinner CD, Gottlieb RL, Criner GJ (2020). Effect of remdesivir vs standard care on clinical status at 11 days in patients with moderate COVID-19: a randomized clinical trial. JAMA.

[5] Yokoyama Y, Briasoulis A, Takagi H, Kuno T (2020). Effect of remdesivir on patients with COVID-19: a network meta-analysis of randomized control trials. Virus Res.

[6] Abdelmajid A, Osman W, Musa H, Elhiday H, Munir W, Al Maslamani MA, Elmekaty EZ (2021). Remdesivir therapy causing bradycardia in COVID-19 patients: two case reports. IDCases.

[7] Maheshwari M, Athiraman H (2021). Bradycardia related to remdesivir during COVID-19: persistent or permanent?. Cureus.

[8] Pan H, Peto R, Henao-Restrepo AM (2021). Repurposed antiviral drugs for COVID-19 - interim WHO solidarity trial results. N Engl J Med.

[9] Beigel JH, Tomashek KM, Dodd LE (2020). Remdesivir for the treatment of COVID-19 - final report. N Engl J Med.

[10] Mulangu S, Dodd LE, Davey RT Jr (2019). A randomized, controlled trial of Ebola virus disease therapeutics. N Engl J Med.

[11] Rafaniello C, Ferrajolo C, Sullo MG (2021). Cardiac events potentially associated to remdesivir: an analysis from the European Spontaneous Adverse Event Reporting System. Pharmaceuticals (Basel).

[12] Bistrovic P, Manola S, Lucijanic M (2022). Bradycardia during remdesivir treatment might be associated with improved survival in patients with COVID-19: a retrospective cohort study on 473 patients from a tertiary centre. Postgrad Med J.

[13] Gubitosa JC, Kakar P, Gerula C (2020). Marked sinus bradycardia associated with remdesivir in COVID-19: a case and literature review. JACC Case Rep.

[14] Pallotto C, Blanc P, Esperti S (2021). Remdesivir treatment and transient bradycardia in patients with coronavirus diseases 2019 (COVID-19). J Infect.

[15] Lucijanic M, Bistrovic P (2022). Remdesivir-associated bradycardia might be a sign of good prognosis in COVID-19 patients. Clin Microbiol Infect.

[16] Choi SW, Shin JS, Park SJ (2020). Antiviral activity and safety of remdesivir against SARS-CoV-2 infection in human pluripotent stem cell-derived cardiomyocytes. Antiviral Res.

[17] Bistrovic P, Lucijanic M (2021). Remdesivir might induce changes in electrocardiogram beyond bradycardia in patients with coronavirus disease 2019-the pilot study. J Med Virol.

[18] Rautaharju PM, Nelson JC, Kronmal RA (2001). Usefulness of T-axis deviation as an independent risk indicator for incident cardiac events in older men and women free from coronary heart disease (the Cardiovascular Health Study). Am J Cardiol.

[19] Kumar S, Arcuri C, Chaudhuri S, Gupta R, Aseri M, Barve P, Shah S (2021). Remdesivir therapy associated with Bradycardia in SARS-CoV2. Clin Cardiol.

[20] Sanchez-Codez MI, Rodriguez-Gonzalez M, Gutierrez-Rosa I (2021). Severe sinus bradycardia associated with remdesivir in a child with severe SARS-CoV-2 infection. Eur J Pediatr.

[21] Varga ZV, Ferdinandy P, Liaudet L, Pacher P (2015). Drug-induced mitochondrial dysfunction and cardiotoxicity. Am J Physiol Heart Circ Physiol.

[22] Elikowski W, Fertała N, Zawodna-Marszałek M (2021). Marked self-limiting sinus bradycardia in COVID-19 patients not requiring therapy in the intensive care unit - case series report. Pol Merkur Lekarski.

